# Tenofovir-associated renal toxicity in a cohort of HIV infected patients in Ghana

**DOI:** 10.1186/s13104-019-4454-2

**Published:** 2019-07-22

**Authors:** Edmund T. Nartey, Raymond A. Tetteh, Barbara A. Yankey, Aukje K. Mantel-Teeuwisse, Hubert G. M. Leufkens, Alexander N. O. Dodoo, Margaret Lartey

**Affiliations:** 1 Centre for Tropical Clinical Pharmacology & Therapeutics, University of Ghana School of Medicine and Dentistry, P. O. Box GP 4236, Accra, Ghana; 2 Utrecht Institute for Pharmaceutical Sciences, Utrecht University, Universiteitsweg 99, 3584 CG Utrecht, The Netherlands; 3 Pharmacy Department, Korle-Bu Teaching Hospital, P.O. Box KB 77, Korle-Bu, Accra, Ghana; 4 Georgia State University, Atlanta, GA USA; 5 Department of Medicine, University of Ghana School of Medicine and Dentistry, P. O. Box GP 4236, Accra, Ghana

**Keywords:** Tenofovir disoproxil fumarate, Renal dysfunction, Creatinine clearance, HIV, ART

## Abstract

**Objective:**

Tenofovir disoproxil fumarate (TDF) is a nucleotide analogue recommended in international HIV treatment guidelines. Purpose of this study was to estimate the long term effects of TDF on renal profile in a cohort of HIV patients in Ghana. Three hundred (300) consecutive HIV-positive patients who initiated TDF-based antiretroviral treatment in 2008 at the Korle-Bu Teaching Hospital were sampled. Creatinine clearance (CrCl) was calculated using the Cockcroft-Gault equation at baseline and renal impairment was defined as CrCl values of 30.0–49.9 mL/min (moderate renal impairment) and < 30 mL/min (severe renal impairment) as per institutional guidelines for renal function test.

**Results:**

Median follow up time was 2.9 years (IQR 2.3–3.4 years). At study endpoint, 63 participants (21.0% [95% CI 6.5–26.1]) recorded CrCl rate below 50 mL/min indicating incident renal impairment, made up of 18.3% moderate renal impairment and 2.3% severe renal impairment. Factors associated with incidence of renal impairment were increasing age, decrease in creatinine clearance rate at baseline, WHO HIV stage III/IV and participants with BMI of < 18.5 kg/m^2^. Patients with identified renal impairment risk factors at ART initiation should be targeted and monitored effectively to prevent renal injury.

## Introduction

Human Immunodeficiency Virus (HIV) and Acquired Immune Deficiency Syndrome (AIDS) are pertinent issues globally, more so in sub-Saharan Africa and in Ghana [1]. The Ghana Aids Commission in 2013 reported the prevalence of HIV to be 1.3% as against 3.6% in 1999 [2]. This significant reduction in prevalence could be attributed to the awareness created through the activities of the National AIDS/STI Control Program (NACP) and the benefits accruing from the life prolonging anti-retroviral drugs (ARV), which also reduces the degree of infectivity of HIV positive patients on subsidized or free highly active antiretroviral therapy (ART). These ARVs are expected to be taken throughout the patient’s life time once the decision to initiate ART is made. ARVs have documented side effects and adverse drug reactions ranging from mild to life threatening ones with their effect being transient or prolonged [3]. Side effects have been reported by patients on these drugs, similar to other chronically administered drugs. Complications related to long-standing HIV infection and treatment, such as the nephrotoxic effects, increase morbidity and mortality of patients.

Tenofovir disoproxil fumarate is an orally bio-available prodrug of tenofovir, an acyclic nucleotide analogue reverse-transcriptase inhibitor (NtRTI), widely used in the treatment of HIV infection and also approved for treatment of Hepatitis B virus infection widely. Tenofovir is preferred in most consolidated ART guidelines [4] in preference to the use of stavudine and zidovudine because of better tolerance, low frequency of adverse events and a once daily dosing combination of tenofovir, lamivudine or emtricitabine and efavirenz [4]. Concerns regarding nephrotoxicity were initially raised by the structural similarity between tenofovir and the nephrotoxic acyclic nucleotide analogues adefovir and cidofovir [5]. Although the incidence of TDF-related kidney dysfunction seems to be low in most settings, the effect of TDF on renal profile in patients starting ART with varying levels of renal function has not been studied previously in our setting. It is against this background that this research was undertaken to study changes in renal function over time in patients on tenofovir based antiretroviral regimen in our patient population at a tertiary hospital in Ghana. We investigated the incidence of renal impairment in HIV positive patients treated with TDF based regimen and identified associated risk factors.

### Main text

## Methods

The Korle Bu Teaching Hospital is a public university tertiary hospital with 2000 beds in Accra, Ghana. The population for this study consisted of HIV positive patients captured in the database used in providing services to patients at the Fevers Unit which is linked to the pharmacy department where patients present for their medications. The study was limited to patients initiated on tenofovir-based regimen within the study period. We undertook a retrospective cohort study of 300 consecutive patients (with baseline creatinine clearance rate of ≥ 50 mL/min) who started tenofovir based regimen from January 2008 with study end-point at December 2013. A clinical research form was used to collect data from patients’ folders. This instrument was developed by the research team, piloted for reliability and validity. The data collection form was pre-tested on 20 folders to remove items not deemed necessary to the expected outcomes. Data of primary interest were demographics, serum creatinine and urea at baseline, weight, tenofovir based regimens, HIV serotyping and CD4 count at baseline. Other information of secondary interest included were co-morbidities and co-medications. Patients were followed up from the study start point of January 2008 until renal impairment, death, or 31st December 2013, whichever came first. Absolute change in creatinine clearance (CrCl) using the Cockcroft-Gault equation was calculated at baseline and as per institutional guidelines for renal function test. Renal impairment was defined as a reduction in CrCl below 50 mL/min (moderate renal impairment) and below 30 mL/min (severe renal impairment). Descriptive and univariate analysis were conducted for demographic, clinical and laboratory characteristics established for the study. Patients’ demography was described using mean ± standard deviation (SD) for continuous variables and percentages for categorical data. Statistical significance of differences between any two groups was tested using appropriate parameters to compare and measure associations. Univariate analysis was done to identify risk factors associated with renal impairment (age, sex, BMI, WHO HIV stage, CD4, TDF regimen type) and reported as relative risk (RR) with 95% confidence interval (CI). A p-value of < 0.05 was considered significant.

## Results

A total of 300 patients with estimated baseline creatinine clearance of > 50 mL/min and initiating TDF containing ART were included in the study. Mean age of the study participants was 39.2 ± 11.1 years and 71.7% (n = 101) were female (Table 1). Baseline BMI of < 18.5 kg/m^2^ (underweight) was present in 17.3% (n = 52) of the study participants. The prevalence of smokers was 3.7% (n = 11) and 13.7% (n = 41) reported that they drank alcohol (Table 1).

**Table 1:** Socio-demographic characteristics of study participants

Characteristics	N = 300 n, %
Age, mean ± SD, years	39.2 ± 11.1
Gender	
Female	215 (71.7)
Male	85 (28.3)
Marital status	
Single	104 (34.7)
Married/cohabiting	163 (54.3)
Divorced/separated/widowed	33 (11.0)
Baseline BMI (kg/m^2^)	
< 18.5 (underweight)	52 (17.3)
18.5–24.9	194 (64.7)
≥ 25.0	54 (18.0)
Educational status	
None	45 (15.0)
Primary/basic	136 (45.3)
Secondary	83 (27.7)
Tertiary	36 (12.0)
Occupation	
Unemployed	33 (11.0)
Self-employed	200 (66.7)
Public/private employment	67 (22.3)
Smoking of tobacco	
Yes	11 (3.7)
No	287 (96.37)
Alcohol use	
Yes	32 (10.7)
No	268 (89.3)
Source of funding	
Self	154 (51.3)
Health insurance	146 (48.7)

Table 2 shows the clinical characteristics of the study participants. Majority of the study participants (97.0%, n = 291) were administered TDF in combination with a Non-nucleoside Reverse Transcriptase Inhibitor (NNRTI), either efavirenz or nevirapine. Median creatinine clearance rate before initiation of TDF containing ART was 76.8 mL/min [Interquartile range (IQR) = 58.3–105.4] and median duration from initiation of TDF containing ART to study end-point or censoring was 2.9 years [IQR = 2.3–3.4]. The most frequent co-morbidities reported were pregnancy (7.3%), anaemia (3.3%) and tuberculosis (3.3%). A total of 40 (13.3%) study participants were on medication other than antiretrovirals and the most frequent co-medications were antibiotics (7.0%) and antihypertensive (4.3%). At study end-point, 63 study participants (21.0%; 95% CI 6.5–26.1) were classified as having renal impairment with CrCl rate < 50.0 mL/min. Seven study participants (2.3%) were further classified as having severe renal impairment with CrCl rate < 30.0 mL/min.

**Table 2:** Clinical characteristics of study participants

Characteristics	N = 300 n, %
Duration on TDF (study endpoint/censoring), median (IQR) years	2.9 [2.3–3.4]
Baseline CrCl rate, median (IQR), mL/min	76.8 [58.3–105.4]
WHO HIV stage	
Stage I	52 (17.3)
Stage II	76 (25.3)
Stage III	134 (44.7)
Stage IV	38 (12.7)
HIV type	
Type I	284 (94.7)
Type II	16 (5.3)
ART regimen administered	
NNRTI-based	291 (97.0)
PI-based	9 (3.0)
Baseline CD_4_ count (cells/mm^3^)	
< 150	134 (44.7)
150–250	58 (19.3)
> 250	108 (36.0)
Adverse event	
Present	47 (15.7)
Absent	253 (84.3)
Co-morbidities^a^	
None	220 (68.0)
Pregnancy	22 (7.3)
Anaemia	10 (3.3)
Tuberculosis	10 (3.3)
Hypertension	9 (3.0)
Malaria	9 (3.0)
Pneumonia	8 (2.7)
Hepatitis	7 (2.3)
Cerebral toxoplasmosis	6 (2.0)
Urinary tract infection	5 (1.7)
Asthma	2 (0.7)
Diabetes	2 (0.7)
Achalasia	1 (0.3)
Deep vein thrombosis	1 (0.3)
Gouty arthritis	1 (0.3)
Psychosis	1 (0.3)
Co-medication^a^	
None	260 (86.7)
Antibiotic	21 (7.0)
Antihypertensive	13 (4.3)
Haematinic	5 (1.7)
Antiallergic	3 (1.0)
Anticoagulant	2 (0.7)
Antidiabetic	2 (0.7)
Antigout	1 (0.3)

ART: Antiretroviral therapy; CrCl: creatinine clearance rate; NNRTI: non-nucleoside reverse transcriptase inhibitor; PI: protease inhibitor; TDF: tenofovir

^a^% May not add up to 100

Table 3 shows the factors associated with incident renal impairment. Age was associated with renal impairment such that for every 1 year increase in age, the risk of renal impairment increased by 4% (RR = 1.04 [95% CI 1.03–1.06]; p < 0.001). Similarly, decreasing baseline creatinine clearance rate was associated with renal impairment, such that for every 1 mL/min decrease in baseline CrCl rate, the risk of incident renal impairment increased by 5% (RR = 1.05 [95% CI 1.04–1.08]; p < 0.001) (Table 3). Patients with WHO HIV staging of either Stage III (RR = 3.78 [95% CI 1.42–10.06]; p < 0.001) or Stage IV (RR = 3.42 [95% CI 1.16–10.09]; p < 0.026) at initiation of TDF-containing ART, were at increased risk of incident renal impairment compared with patients of Stage I (Table 3).

**Table 3:** Factors associated with renal impairment in patients attending HIV clinic at the KBTH

Characteristic	Renal impairment N = 63 n, %	No renal impairment N = 237 n, %	Relative risk [95% CI]	p-value
Age, mean ± SD, years	45.3 ± 12.15	37.6 ± 10.3	1.04 [1.03–1.06]	*<* *0.001*
Baseline CrCl rate, median (IQR), mL/min	55.7 [52.4–66.1]	85.7 [66.9–113.2]	0.95 [0.93–0.96]	*< 0.001*
Gender				
Male	18 (28.6)	67 (28.3)	1.01 [0.62–1.64]	0.962
Female	45 (71.4)	170 (71.7)	1.00	
Smoke tobacco				
Yes	2 (3.2)	9 (3.8)	0.86 [0.24–3.06]	0.810
No	61 (96.8)	226 (96.2)	1.00	
Alcohol use				
Yes	4 (6.4)	28 (11.8)	0.57 [0.22–1.46]	0.240
No	59 (93.6)	209 (88.2)	1.00	
WHO HIV stage				
Stage IV	4 (6.3)	48 (20.2)	3.42 [1.16–10.09]	*0.026*
Stage III	10 (15.9)	66 (27.9)	3.78 [1.42–10.06]	*0.008*
Stage II	39 (61.9)	95 (40.1)	1.71 [0.57–5.16]	0.341
Stage I	10 (15.9)	28 (11.8)	1.00	
HIV type				
Type II	2 (3.2)	14 (5.9)	0.58 [0.16–2.17]	0.420
Type I	61 (96.8)	223 (94.1)	1.00	
ART regimen administered				
PI-based	2 (3.2)	7 (3.0)	1.06 [0.31–3.67]	0.927
NNRTI-based	61 (96.8)	230 (97.0)	1.00	
Baseline CD_4_ count (cells/mm^3^)				
< 150	30 (47.6)	104 (43.9)	1.42 [0.83–2.44]	0.200
150–250	16 (25.4)	42 (17.7)	1.75 [0.96–3.20]	0.068
> 250	17 (27.0)	91 (38.4)	1.00	
Presence of co-morbidity				
Yes	20 (31.7)	60 (25.3)	1.28 [0.80–2.04]	0.299
No	43 (68.3)	177 (74.7)	1.00	
On co-medication				
Yes	12 (19.1)	28 (11.8)	1.53 [0.90–2.61]	0.119
No	51 (80.9)	209 (88.2)	1.00	
Baseline BMI (kg/m^2^)				
< 18.5 (underweight)	27 (42.8)	25 (10.5)	3.87 [2.49–6.03]	*< 0.001*
≥ 25.0	10 (15.9)	44 (18.6)	1.38 [0.71–2.68]	0.340
18.5–24.9	26 (41.3)	168 (70.9)	1.00	
Adverse event				
Present	12 (19.1)	35 (14.8)	1.27 [0.73–2.19]	0.397
Absent	51 (80.9)	202 (85.2)	1.00	

Italic values indicate significance of p-value at p < 0.05

ART: Antiretroviral therapy; BMI: body mass index; CI: confidence interval; CrCl; creatinine clearance; IQR: interquartile range; NNRTI: non-nucleoside reverse transcriptase inhibitor; PI: protease inhibitor; SD: standard deviation

## Discussion

We found that approximately 1 out of 5 patients on tenofovir based regimen from the Korle Bu Teaching Hospital HIV Clinical Care program experienced development of renal impairment over the 5 years period of this study. It is worth noting that of these about 2.3% developed severe renal impairment. Factors associated with the incidence of renal impairment were older age, lower CrCl at baseline, WHO HIV stages III and IV compared to those with stage I and baseline BMI below 18.5 kg/m^2^ (underweight).

The Incidence of renal impairment after initiation of TDF based regimens is varied across studies [6–9], but tended to be lower than the incidences observed in this study. This could be due to different study method approaches used and/or varying renal impairment incidences in different populations. However, a Japanese retrospective study of 493 patients initiated on TDF based regimen reported similar incidence of declining renal function [10], comparable with this study. The clinical implication is not certain but it is proven that patients on tenofovir based regimen tend to develop decreases in renal performance as compared to those on non-tenofovir based regimens [11]. Lower BMI of < 18.5 kg/m^2^ (underweight) was evident in 17% of the participants at baseline and it was found to be associated with incident renal impairment which is similar to earlier reports [10, 12]. Although findings from this study indicate no association between type of TDF regimen administered (TDF-based regimen with protease inhibitors lopinavir/ritonavir or TDF-based regimen with non-nucleoside inhibitors efavirenz or nevirapine) and renal impairment, other studies reported that the degree of renal function decline was more frequent and more serious in TDF-based regimen with protease inhibitors lopinavir/ritonavir than TDF-based regimen with non-nucleoside inhibitors (efavirenz or nevirapine) [13, 14]. The number of patients on TDF-based protease inhibitor regimen in our study was however low and therefore could account for the lack of association between type of TDF-based regimen and renal impairment. Fifty-seven percent (57%) of participants were found to have WHO Stage III and IV disease and this was found to be associated with declining renal performance. Literature supports this finding as worsening HIV disease results in various opportunistic infections that worsen kidney performance [15]. Older age was established to be associated with renal impairment in our study analysis and this is consistent with other studies which associated older age with low baseline CrCl [12, 13, 15].

The study observed that patients classified at baseline as WHO stages 3 and 4 and those with lower BMI as specified in the study report should have baseline renal assessment done before the initiation of TDF based therapy. TDF based regimen is the preferred antiretroviral therapy option for countries in sub-Saharan Africa and with the recent 90/90/90 target declaration by WHO, the question of whether long term usage will pose major problems for the more African HIV positive patients who are likely to be initiated on TDF in resource limited settings is paramount. The determination of about 20% renal impairment in this study gives an indication of possible problems in future, more so with the WHO recommendation of “Treat All” [16, 17], irrespective of CD4 count and laboratory monitoring of baseline renal performance. This finding evidently calls for prudent monitoring of all patients on TDF based regimen with CrCl below the normal range of > 80 mL/min [8, 18]. The incidence of renal impairment comes against a background of determined prevalence of 10% renal impairment in all medical admissions as reported in the Korle-Bu Teaching Hospital’s 2012 Annual Report [19]. In this context, the issue of long-term nephrotoxicity is important and much can be achieved in reducing this risk by health professionals observing operational norms as recommended by protocols and guidelines provided for use. On a large scale as pertains in the National HIV Programme, lack of adequate and skilled human resources, equipment and reagents for checking of creatinine clearance remains a hurdle to contend with. It is recommended therefore that simple and feasible renal screening tests like dipstick testing for urine protein be implemented [20]. Patients with positive urine protein testing on dipstick can then be offered serum creatinine testing. This offers a cheaper and a quicker screening tool which can easily be deployed in all rural and deprived areas for initial screening to eliminate patients with the potential to develop renal discomfort when initiated on TDF based regimen. Measuring renal function carefully to assess possibility of renal disease before prescribing TDF based regimen for the higher risk patients as indicated by the findings of this study should be a yardstick for initiating ART irrespective of the” treat all “policy since our primary concern is to protect patients from renal injury.

## Conclusion

The use of TDF based regimen as first line ART regimen in Ghana and most African countries is justified by its beneficial attributes. However, the incidence of renal impairment of 1 in every 5 patients with 3% developing severe renal impairment on TDF-based ART as determined in this study supports the argument of requesting for laboratory support before initiating TDF despite our resource challenged circumstances. Additionally, all patients started with TDF based regimen in response to the Treat All or the 90/90/90 target by 2020 policy initiative by WHO should be monitored in time while on ART to prevent untoward effects.

## Limitations

- The unavailability of creatinine and urea recordings regularly is considered a limitation to this study. We had to use available recordings as and when available in the medical folders and the database.
- The sample size of 300 limited us in assessing the association between the various TDF-based regimen types and renal impairment and also conduct a multiple regression analysis of the factors associated with renal impairment. As such, the observed factors may not be independently associated with renal impairment since potential confounders/covariates were not controlled for statistically in the analysis.

## Data Availability

The datasets generated and/or analysed during the current study are not publicly available due to the fact that they are confidential patient data of HIV-positive individuals but are available from the corresponding author on reasonable request.

## Abbreviations

AIDS: Acquired Immune Deficiency Syndrome
ARV: anti-retroviral drug
ART: highly active antiretroviral therapy
BMI: body mass index
CrCl: creatinine clearance
HIV: Human Immunodeficiency Virus
IQR: interquartile range
KBTH: Korle-Bu Teaching Hospital
NACP: National AIDS/STI Control Program
NNRTI: non-nucleoside reverse transcriptase inhibitor
NtRTI: nucleotide reverse-transcriptase inhibitor
TDF: tenofovir disoproxil fumarate
WHO: World Health Organisation

## References

[1] World Health Organisation (WHO) (2013). Global update on HIV treatment 2013: results, impact and opportunities.

[2] Ghana AIDS Commisssion (GAC). Ghana’s progress report on the United Nations General Assembly Special Session (UNGASS) declaration of commitment on HIV and AIDS Geneva, Switzerland: GAC; 2013.

[3] Eluwa GI, Badru T, Agu KA, Akpoigbe KJ, Chabikuli O, Hamelmann C (2012). Adverse drug reactions to antiretroviral therapy (ARVs): incidence, type and risk factors in Nigeria. BMC Clin Pharmacol.

[4] Estrella M, Moosa M, Nachega J (2014). Risk and benefits of tenofovir in the context of kidney dysfunction in sub-Sarahan Africa. CID.

[5] Gallant JE, Staszewski S, Pozniak AL, DeJesus E, Suleiman JM, Miller MD (2004). Efficacy and safety of tenofovir DF vs stavudine in combination therapy in antiretroviral-naive patients: a 3-year randomized trial. JAMA.

[6] Horberg M, Tang B, Towner W, Silverberg M, Bersoff-Matcha S, Hurley L (2010). Impact of tenofovir on renal function in HIV-infected, antiretroviral-naive patients. J Acquir Immune Defic Syndr.

[7] Calza L, Trapani F, Tedeschi S, Piergentili B, Manfredi R, Colangeli V (2011). Tenofovir-induced renal toxicity in 324 HIV-infected, antiretroviral-naive patients. Scand J Infect Dis.

[8] Chua AC, Llorin RM, Lai K, Cavailler P, Law HL (2012). Renal safety of tenofovir containing antiretroviral regimen in a Singapore cohort. AIDS Res Ther.

[9] De Beaudrap P, Diallo MB, Landman R, Gueye NF, Ndiaye I, Diouf A (2010). Changes in the renal function after tenofovir-containing antiretroviral therapy initiation in a Senegalese cohort (ANRS 1215). AIDS Res Hum Retroviruses.

[10] Nishijima T, Komatsu H, Gatanaga H, Aoki T, Watanabe K, Kinai E (2011). Impact of small body weight on tenofovir-associated renal dysfunction in HIV-infected patients: a retrospective cohort study of Japanese patients. PLoS ONE.

[11] Cooper RD, Wiebe N, Smith N, Keiser P, Naicker S, Tonelli M (2010). Systematic review and meta-analysis: renal safety of tenofovir disoproxil fumarate in HIV-infected patients. Clin Infect Dis.

[12] Madeddu G, Bonfanti P, De Socio GV, Carradori S, Grosso C, Marconi P (2008). Tenofovir renal safety in HIV-infected patients: results from the SCOLTA Project. Biomed Pharmacother.

[13] Goicoechea M, Liu S, Best B, Sun S, Jain S, Kemper C (2008). Greater tenofovir-associated renal function decline with protease inhibitor-based versus nonnucleoside reverse-transcriptase inhibitor-based therapy. J Infect Dis.

[14] Quesada PR, Esteban LL, Garcia JR, Sanchez RV, Garcia TM, Alonso-Vega GG (2015). Incidence and risk factors for tenofovir-associated renal toxicity in HIV-infected patients. Int J Clin Pharm.

[15] Kamkuemah M, Kaplan R, Bekker LG, Little F, Myer L (2015). Renal impairment in HIV-infected patients initiating tenofovir-containing antiretroviral therapy regimens in a Primary Healthcare Setting in South Africa. Trop Med Int Health.

[16] World Health Organisation (WHO) (2015). Consolidated guideliness for treatment of persons living with HIV.

[17] National AIDS Control Program (NACP). Guidelines for antiretroviral therapy in Ghana. Accra; 2014.

[18] Bygrave H, Kranzer K, Hilderbrand K, Jouquet G, Goemaere E, Vlahakis N (2011). Renal safety of a tenofovir-containing first line regimen: experience from an antiretroviral cohort in rural Lesotho. PLoS ONE.

[19] Korle-Bu Teaching Hospital (KBTH). Korle-Bu teaching hospital annual report-2012. Accra. Ghana: KBTH 2012.

[20] Karras DL, Helpern KL, Riley LJ, Hughes L, Gaughan JP (2002). Urine dipstick as screening test for serum creatinine elevation in emergency department patients with severe hypertension. Acad Emerg Med.

